# Significant Functional Differences Between Dopamine D_4_ Receptor Polymorphic Variants Upon Heteromerization with α_1A_ Adrenoreceptors

**DOI:** 10.1007/s12035-023-03476-8

**Published:** 2023-07-18

**Authors:** Patricia Homar-Ruano, Ning-Sheng Cai, Verònica Casadó-Anguera, Vicent Casadó, Sergi Ferré, Estefanía Moreno, Enric I. Canela

**Affiliations:** 1 Department of Biochemistry and Molecular Biomedicine, Faculty of Biology, University of Barcelona, Barcelona, Spain; 2 Institute of Biomedicine of the University of Barcelona (IBUB), Barcelona, 08028 Spain; 3 National Institute On Drug Abuse, Intramural Research Program, National Institutes of Health, Department of Health and Human Services, Baltimore, MD 21224 USA

**Keywords:** Receptor heteromers, α_1A_ adrenoreceptor, Dopamine D_4_ receptor, Polymorphic variants, ADHD, Cortex, Striatum

## Abstract

The functional role of the dopamine D_4_ receptor (D_4_R) and its main polymorphic variants has become more evident with the demonstration of heteromers of D_4_R that control the function of frontal cortico-striatal neurons. Those include heteromers with the α_2A_ adrenoceptor (α_2A_R) and with the D_2_R, localized in their cortical somato-dendritic region and striatal nerve terminals, respectively. By using biophysical and cell-signaling methods and heteromer-disrupting peptides in mammalian transfected cells and rat brain slice preparations, here we provide evidence for a new functionally relevant D_4_R heteromer, the α_1A_R-D_4_R heteromer, which is also preferentially localized in cortico-striatal glutamatergic terminals. Significant differences in allosteric modulations between heteromers of α_1A_R with the D_4.4_R and D_4.7_R polymorphic variants could be evidenced with the analysis of G protein-dependent and independent signaling. Similar negative allosteric modulations between α_1A_R and D_4_R ligands could be demonstrated for both α_1A_R-D_4.4_R and α_1A_R-D_4.7_R heteromers on G protein-independent signaling, but only for α_1A_R-D_4.4_R on G protein-dependent signaling. From these functional differences, it is proposed that the D_4.4_R variant provides a gain of function of the α_1A_R-mediated noradrenergic stimulatory control of cortico-striatal glutamatergic neurotransmission, which could result in a decrease in the vulnerability for impulse control-related neuropsychiatric disorders and increase in the vulnerability for posttraumatic stress disorder.

## Introduction

The functional role of the dopamine D_4_ receptor (D_4_R) and its polymorphic variants in the brain is beginning to be understood with the realization that it can also be a target for norepinephrine and with the discovery of heteromers of D_4_Rs with other dopamine receptors and with several adrenoceptor subtypes (for review, see ref. [1]). This is exemplified in the pineal gland, which together with the retina and the frontal cortex are the main localizations of the D_4_R in the brain. In the pinealocytes, which are the melatonin-producing cells, D_4_Rs experience a significant circadian expression, with highest levels at the end of the dark period [2]. This is associated with an increased formation of heteromers of D_4_Rs with α_1B_ and β_1_ adrenoceptors [3]. At darkness, norepinephrine, by also activating D_4_Rs promotes an allosteric inhibition of α_1B_R and β_1_R signaling in the respective heteromers, which results in a reduction in the synthesis and release of melatonin [3].

In the prefrontal cortex, D_4_Rs are expressed by GABAergic interneurons and by glutamatergic pyramidal neurons, both in their cortical somato-dendritic region and striatal nerve terminals [1]. Several studies have shown that D_4_Rs play a significant role in the modulation of the frontal-cortico-striatal neuronal function (for review, see ref. [1]). This role also depends on heteromerization, with α_2A_ adrenoceptors (α_2A_Rs) in the cortical perisomatic region [4], and with dopamine D_2_ receptors (D_2_Rs) in the cortico-striatal terminals [5, 6]. The understanding of the functional role of these D_4_R heteromers has been concomitant to the finding of different properties of the major human D_4_R gene (*DRD4*) polymorphic variants.

The human D_4_R gene (*DRD4*) displays a high number of polymorphisms in its coding sequence. The most extensive polymorphism is found in exon 3, a region that encodes the third intracellular loop (3IL) of the receptor [7–9]. This polymorphism includes a variable number of tandem repeats of a 48-base pair sequence. The most common polymorphisms contain 4 or 7 repeats (with allelic frequencies of about 60% and 20%, respectively), which encode a D_4_R with the respective number of repeats of a proline-rich sequence of 16 amino acids (D_4.4_R and D_4.7_R) [7–9]. Importantly, *DRD4* polymorphisms have been associated with personality traits that constitute endophenotypes for impulse control-related neuropsychiatric disorders [1], with the most consistent associations found between the gene encoding D_4.7_R and attention-deficit hyperactivity disorder (ADHD) [8, 10–12] and substance use disorders (SUDs) [13].

Notably, clear qualitative differences in the functional and pharmacological properties of these polymorphic variants have only been observed when analyzing those properties upon heteromerization with α_2A_Rs and D_2_Rs. Heteromerization with D_4.7_R, but not D_4.4_R, significantly increases the constitutive activity of the D_2_R and the signaling potency of dopamine, as compared with non-heteromerized D_2_R [14]. This provided a biochemical correlate of a gain of function of the D_2_R-D_4.7_R heteromers localized in glutamatergic terminals as mediators of an inhibition of cortico-striatal neurotransmission, which was demonstrated with immunohistochemical and in vivo optogenetic-microdialysis experiments in D_4.7_R knock-in mice expressing a humanized D_4_R with the 3IL of the human D_4.7_R [5].

Similarly, heteromerization of α_2A_R with D_4.7_R, but not D_4.4_R, significantly increases the signaling potency of norepinephrine for the α_2A_R [4]. On the other hand, D_4.4_R, but not D_4.7_R activation, allosterically inhibits α_2A_R-mediated signaling in their respective heteromers [4], comparatively to the negative allosteric inhibition of α_1B_R and β_1_R in the respective heteromers in the pineal gland [3]. We proposed that the main functional output of the activation of cortical α_2A_R-D_4.7_R heteromers is a decrease in the excitability of glutamatergic pyramidal neurons, which should provide an additional gain of function of D_4_R in its inhibitory control of frontal cortico-striatal neurotransmission [1].

Interestingly, another adrenoceptor, the α_1A_ receptor (α_1A_R) is also expressed by frontal cortico-striatal pyramidal neurons and predominantly localized in cortico-striatal terminals, but also on its perisomatic region [15, 16]. Although, also in the frontal cortex α_1A_Rs are preferentially localized presynaptically in glutamatergic terminals [15]. Activation of α_1A_Rs in the prefrontal cortex and the striatum leads to an increased activity and glutamate release by the pyramidal cortico-striatal neuron [15, 16]. The α_1A_R has lower affinity than the α_2A_R and has been conceptualized as a receptor that mediates the effect of stress-induced norepinephrine release, with possible implications for the pathophysiology and treatment of posttraumatic stress disorder (PTSD) [17]. The ability of D_4_Rs to form heteromers with several adrenoceptor subtypes and their clear potential colocalization with α_1A_Rs in the pyramidal cortico-striatal glutamatergic neuron, led us to investigate the possible existence of functional α_1A_R-D_4_R heteromers in vitro and in vivo, as well as the possible pharmacological differences that would depend on the D_4.4_R and D_4.7_R polymorphic variants.

## Materials and Methods

### Cell Culture and Transfection

Human Embryonic Kidney-293 T (HEK-293 T) cells and two previously characterized HEK‐293 T cell lines with tetracycline inducible expression of the D_4_R polymorphic variants D_4.4_R or D_4.7_R were used [4]. Cells were grown in Dulbecco’s Modified Eagle’s Medium (DMEM) supplemented with 5% Fetal Bovine Serum (FBS) and kept in an incubator at 37 °C and 5% CO_2_. The inducible HEK‐293 T cells were obtained with the Flp‐In T‐Rex system and were maintained with hygromycin 50 μg/ml and blasticidin 15 μg/ml and the D_4_R variant expression was induced for 18‐24 h with administration of tetracycline (250 ng/ml). Cells were transiently transfected with cDNA corresponding to the specific fused or non-fused receptors, G protein subunits or β-arrestin-2 using polyethyleneimine (Sigma-Aldrich, Cerdanyola del Vallés, Spain) or Lipofectamine 2000 (in calcium release experiments). All experiments were performed 48 h after transfection.

### DNA Constructs

The cDNAs of the human D_4.4_R, D_4.7_R, α_1A_R and α_2A_R expressed in the pcDNA3.1 vector were amplified without its stop codon using sense and antisense primers harbouring BamHI and EcoRI restriction sites and subcloned into pRluc-N1 (Rluc) or pEYFP-N1 (YFP) vectors. For BiLC assays, human α_1A_R, D_4.4_R and D_4.7_R expressing the amino acid residues 1–229 (nRluc) or 230–311 (cRluc) of the Rluc8 variant were subcloned into pcDNA3.1. For β-arrestin-2 recruitment assay, α_1A_R-YFP, non-fused D_4.4_R and D_4.7_R, and β-arrestin-2 fused to pRluc-N1 were used. For G_α_ protein activation assays the following human constructs were used: non-fused D_4.4_R, D_4.7_R and α_1A_R, G_αi1_-Rluc (Rluc8 variant) with Rluc inserted at position 91, G_αs_-Rluc with Rluc inserted at position 67, G_αq_-Rluc with Rluc inserted at position 97, non-fused G_β1_, and G_γ2_ fused to YFP (mVenus variant) at the N terminus.

### TAT‐TM Peptides

Synthetic peptides with the amino acid sequences of human D_4_Rs and α_1_R transmembrane domains (TMs) (Peptide Synthesis Facility, University Pompeu Fabra, Barcelona) fused to a peptide derived from the HIV-transactivator of transcription (TAT: YGRKKRRQRRR) were used as receptor heteromer-disrupting molecules. The cell-penetrating TAT peptide binds to the phosphatidylinositol‐(4,5)-bisphosphate found on the inner surface of the membrane, allowing the right orientation of the peptide when inserted in the plasma membrane [18]. TM4, TM5, TM6 and TM7 peptides of the D_4_Rs and α_1_R were chosen since TM4, TM5 and/or TM6 are often involved in the interface of GPCR heteromers, and TM7 is often used as a negative control [4, 19–22]. HIV-TAT peptide was then fused to the N‐terminus of TM4 and TM6 and to the C-terminus of TM5 and TM7. The amino acid sequences were:

- **TAT**‐TM4 de D_4_R: **RRRQRRKKRGY**GSRRQLLLIGATWLLSAAVAAPVLCGL.
- TM5‐**TAT** de D_4_R: YVVYSSVCSFFLPCPLMLLLYWATF**YGRKKRRQRRR.**
- **TAT**‐TM6 de D_4_R: **RRRQRRKKRGY**VLPVVVGAFLLCWTPFFVVHI.
- TM7‐**TAT** de D_4_R: LVSAVTWLGYVNSALNPVIYTVFNAYGRKKRRQRRR.
- **TAT**‐TM4 de α_1_R: **RRRQRRKKRGY**LMALLCVWALSLVISIGPLFGWRQ.
- TM5‐**TAT** de α_1_R: PGYVLFSALGSFYLPLAIILVMYC**YGRKKRRQRRR.**
- **TAT**‐TM6 de α_1_R: **RRRQRRKKRGY**LGIVVGCFVLCWLPFFLVMPIGSF.
- TM7‐**TAT** de α_1_R: TVFKIVFWLGYLNSCINPIIYPCS**YGRKKRRQRRR.**

### BRET and BiLC Assays

For the bioluminescence resonance energy transfer (BRET) assays, HEK-293 T cells were transiently co‐transfected with a constant amount of the cDNA encoding the Rluc-fused receptor and increasing amounts of cDNA encoding the YFP-fused receptor. The cell medium was removed and replaced with 0.1% glucose supplemented Hank’s Balanced Salt Solution (HBSS) buffer (140 mM NaCl, 5 mM KCl, 1.2 mM CaCl_2_, 0.4 mM MgSO_4_·7H_2_O, 0.5 mM MgCl_2_·6H_2_O, 0.3 mM Na_2_HPO_4_, 0.4 mM KH_2_PO_4_ and 4 mM NaHCO_3_) and cells were collected. The protein concentration in collected intact cell preparations was determined using the Bradford assay kit (Bio-rad; Munich, Germany), with bovine serum albumin dilutions as standards. For fluorescence quantification, 20 μg of protein were plated in 96-well black and transparent bottom microplates and fluorescence measured as the emission at 530 nm after 500 nm excitation in a Mithras LB 940 (Berthold Technologies, Bad Wildbad, Germany). Separately, for BRET measurements 20 μg of protein were plated in 96-well white microplates and 5 μM of Coelenterazine H (Invitrogen) was added one minute before BRET signal acquisition using the Mithras LB 940 reader. BRET signal was determined as the ratio of the light emitted by YFP (530 nm) over that emitted by coelenterazine H (485 nm). Rluc expression was also quantified by reading luminescence 10 min after the addition of coelenterazine H. Net BRET was defined as ((long-wavelength emission)/(short-wavelength emission)) – Cf, where Cf corresponds to ((long-wavelength emission)/(short-wavelength emission)) of the Rluc protein expressed individually. BRET is expressed as milliBRET units (mBU). Data were fitted to a nonlinear regression equation, assuming a single‐phase saturation curve, with GraphPad Prism 9 software. For bimolecular luminescent complementation (BiLC) assays, cells were co-transfected with the cDNA encoding the receptors of interest fused to Rluc hemiproteins (nRluc and cRluc). After 48 h, cells were treated or not with the indicated TAT-TM peptides (2 μM) for 4 h at 37 °C. The quantification of the receptor-reconstituted Rluc expression was measured at 485 nm after 10 min of adding coelenterazine H. Cells expressing the receptor fused to one hemiprotein showed similar luminescence levels to nontransfected cells.

### G-Protein Activation and β-Arrestin-Recruitment BRET Assays

Variations of BRET assays were also performed to detect ligand-induced activation of distinct subtypes of G_α_ protein and β-arrestin recruitment. For G-protein activation assay, expression vectors coding different Rluc-fused G_α_ protein subunits and YFP-fused G_γ2_ protein were co-transfected with the receptor or receptors of interest and non-fused G_β1_ constructs. For β-arrestin recruitment, Rluc (Rluc8 variant)-fused β-arrestin-2, α_1A_R-YFP and non-fused D_4.4_R and D_4.7_R constructs were co-transfected for BRET detection. As previously reported [14], cells were harvested, washed, and resuspended in 0.1% glucose supplemented Hank’s Balanced Salt Solution (HBSS) buffer. Approximately 200,000 intact cells/well were distributed in 96-well plates, and 5 μM coelenterazine H (substrate for luciferase) was added to each well. Two minutes after the addition of coelenterazine H, agonists were added to each well, whereas antagonists were added 10 min before the addition of the agonist. The acceptor fluorescence was quantified (excitation at 500 nm and emission at 540 nm for 1-s recordings) in Mithras LB940 (Berthold Technologies, Bad Wildbad, Germany) to confirm the constant expression levels across experiments. In parallel, the BRET signal from the same batch of cells was determined as the ratio of the light emitted by mVenus/YFP (530 nm) over that emitted by Rluc (485 nm) in PHERAstar Flagship microplate reader (BMG Lab technologies, Offenburg, Germany). The ligand induced events were calculated as the BRET change (BRET ratio for the corresponding drug minus the BRET ratio in the absence of the drug) observed after the addition of the ligands. BRET curves were analyzed by nonlinear regression using GraphPad software. All ligands tested are from Tocris Bioscience; Bristol, UK.

### cAMP Accumulation Assay

The HEK-293 T cell lines with inducible expression of D_4.4_R or D_4.7_R were transfected with non-fused α_1A_ receptor. Two hours before initiating the assay, cell culture medium was substituted by serum-free medium. Cells were then detached and resuspended in serum-starved medium containing 50 μM zardaverine, 0.1% BSA and 5 mM HEPES and were plated in 384-well microplates (1500 cells/well) and treated with the corresponding ligands. Cells were then pre-treated with the antagonists or vehicle for 15 min and then stimulated with agonists also for 15 min. In case of Gi-mediated inhibitory signaling, cells were stimulated for 15 min with forskolin after agonists treatment. Intracellular cAMP production was quantified by homogeneous time-resolved fluorescence (HTRF) energy transfer method using the Lance Ultra cAMP kit (PerkinElmer, Waltham, Massachusetts, US). Fluorescence readings at 665 nm were performed on a PHERastar Flagship Microplate Reader (BMG Labtech, Ortenberg, Germany) equipped with an HTRF optical module.

### Intracellular Calcium Release

To determine intracellular calcium free concentration, HEK-293 T cells were co-transfected with cDNA encoding the receptor or receptors of interest and 3 μg of the GCaMP6 calcium sensor. Cells were harvested, washed, and resuspended in Mg^2+^-free Locke’s buffer (154 mM NaCl, 5.6 mM KCl, 3.6 mM NaHCO_3_, 2.3 mM CaCl_2_, 5.6 mM glucose, 5 mM HEPES, pH 7.4) supplemented with 10 μM glycine. The protein concentration in collected intact cell preparations was determined using the Bradford assay kit (Bio-rad; Munich, Germany), using bovine serum albumin dilutions as standards. Then, 40 µg of protein were plated in 96-well black, clear-bottom microplates and treated with the desired ligands. Fluorescence emission intensity of the GCaMP6 sensor was recorded for 150 s (30 flashes/well) at 515 nm upon excitation at 488 nm on an EnSpire® Multimode Plate Reader (Perkin Elmer; Wellesley, MA, United States).

### Brain Slice Preparation

Male Sprague Dawley rats (2 months old; from the animal facility of the Faculty of Biology, University of Barcelona) were used. The animals were housed two per cage and kept on a 12 h dark/light cycle with food and water available ad libitum, and experiments were performed during the light cycle. All procedures were approved by the Ethical Committee for Animal Use of University of Barcelona (OB 408/18 and OB 409/18). Animals were killed by decapitation under 4% isoflurane anesthesia, and brains were rapidly removed, placed in ice-cold oxygenated (O_2_/CO_2_, 95%/5%) Krebs–HCO_3_^−^ buffer (containing [in mM]: 124 NaCl, 4 KCl, 1.25 KH_2_PO_4_, 1.5 MgCl_2_, 1.5 CaCl_2_, 10 glucose, and 26 NaHCO_3_, pH 7.4), and sliced coronally at 4 °C using a brain matrix (Zivic Instruments). Slices of the prefrontal cortex or striatum (500 μm thick) were dissected at 4 °C in Krebs–HCO_3_^−^ buffer; each slice was transferred into an incubation tube containing 1 ml of ice-cold Krebs–HCO_3_^−^ buffer. The temperature was raised to 23 °C, and after 30 min the medium was replaced by 2 ml of fresh buffer or TM peptides prepared in Krebs–HCO_3_^−^ buffer at 4 µM. Slices were incubated under constant oxygenation (O_2_/CO_2_, 95%/5%) at 30 °C for 4 h in an Eppendorf Thermomixer (5 Prime, Boulder, Colorado, USA). Then, the medium was replaced with 200 μl of fresh buffer and incubated for 30 min before the addition of any ligand. After incubation with the corresponding ligands, the solution was discarded, and slices were frozen on dry ice and stored at − 80 °C. The tissue was lysed by the addition of ice-sold lysis buffer and treated as described below for HEK‐293 T cells for ERK1/2 phosphorylation determination**.**

### ERK1/2 Phosphorylation Assay

HEK‐293 T cells were co-transfected with α_1A_R and D_4.4_R or D_4.7_R. The day of the experiment, the culture medium was substituted by serum-starved medium 4 h before treatment with the ligands of interest for 10 min in case of antagonists and 7 min for agonists, at 37 °C and in humid atmosphere. Then, cells were placed in ice to stop the metabolism and cells were washed with ice‐cold PBS. Successively, ice-cold lysis buffer (50 mM Tris–HCl pH 7.4, 50 mM NaF, 150 mM NaCl, 45 mM-glycerophosphate, 1% Triton X-100, 20 μM phenylarsine oxide, 0.4 mM NaVO_4_ and protease inhibitor) was added. The cellular debris was removed by centrifugation at 13,000 g for 10 min at 4 °C, and the protein levels were quantified by the bicinchonic acid method, using bovine serum albumin dilutions as standard. The samples were stored and then processed for immunoblotting as described below.

### Western Blotting

Determination of protein levels by immunoblotting was carried out in transfected cells or in brain slices to determine the level of ERK1/2 phosphorylation. Equivalent amounts of cell protein were separated by polyacrylamide gel electrophoresis on denaturing conditions (10% SDS). Proteins were transferred into polyvinylidene fluoride membranes and then treated with odyssey blocking buffer (LI‐COR Biosciences, Lincoln, Nebraska) for 1 h. Primary antibodies mixture of a mouse anti– phospho‐ERK1/2 antibody (1:2500; Sigma‐Aldrich) and rabbit anti‐ERK1/2 antibody (1:40,000; Sigma‐Aldrich) were added and kept over-night at 4 °C. After removal of the primary antibodies the 42‐ and 44‐kDa bands corresponding to ERK1 and ERK2 were visualized by the addition of a mixture of IRDye800 (anti‐mouse) antibody (1:10,000; Sigma‐Aldrich) and IRDye 680 (anti‐rabbit) antibody (1:10,000; Sigma‐Aldrich) for 2 h and scanned by the Odyssey infrared scanner (LICOR Biosciences). Band densities were quantified using the scanner software and exported to Excel (Microsoft, Redmond, WA). The level of phosphorylated ERK1/2 isoforms was normalized for differences in loading using the total ERK1/2 protein band intensities.

### In situ Proximity Ligation Assay

Rat brain slices were fixed by immersion in 4% PFA solution for 1 h at 4 °C. Samples were then washed in 50 mM Tris‐HCl, 0.9% NaCl pH 7.8 buffer (TBS), cryopreserved in a 30% sucrose solution for 48 h at 4 °C, and stored at ‐20 °C until sectioning. 20 μm‐thick slices were cut coronally (frontal to bregma AP = 0) on a freezing cryostat (Leica Jung CM‐3000), mounted on slide glass and frozen at ‐20 °C until use. To perform the PLA, slices were thawed at 4 °C, washed in PBS, permeabilized with PBS containing 0.01% Triton X‐100 for 10 min, and successively washed with PBS. Heteromers were detected using the Duolink II in situ PLA detection Kit (Sigma-Aldrich) and following the instructions of the supplier. To detect α_1A_R‐D_4_R complexes, a mixture of equal amounts of mouse anti‐α_1A_R antibody (Thermo Scientific, Fremont, California) and goat anti‐D_4_R (sc‐1439) (Santa Cruz Biotechnology, Santa Cruz, California) antibody was used. Samples were further incubated with anti‐mouse plus and anti‐goat minus PLA probes. Slices were mounted using DAPI-containing mounting medium and observed in a Leica SP2 confocal microscope (Leica Microsystems, Mannheim, Germany) equipped with an apochromatic 63X oil‐immersion objective (N.A. 1.4), and a 405 nm and a 561 nm laser line. For each field of view, a stack of two channels (one per staining) and 9 to 15 Z stacks with a step size of 1 μm were acquired. Images were opened and processed with Image J software (National Institutes of Health, Bethesda, MD). Quantification of cells containing one or more red dots versus total cells (blue nucleus) was determined by using the Fiji package (https://fiji.sc/). Nuclei and red spots were counted on the maximum projections of each image stack. After getting the projection, each channel was processed individually. The blue nuclei were segmented by filtering with a median filter, subtracting the background, enhancing the contrast with the Contrast Limited Adaptive Histogram Equalization (CLAHE) plug‐in, and finally applying a threshold to obtain the binary image and the regions of interest (ROIs) around each nucleus. Red spot images were also filtered and thresholded to obtain the binary images. Red spots were counted in each of the ROIs obtained in the nuclei images.

## Results

### In vitro Identification of α_1A_R-D_4.4_R and α_1A_R-D_4.7_R Heteromers

**Fig. 1:**
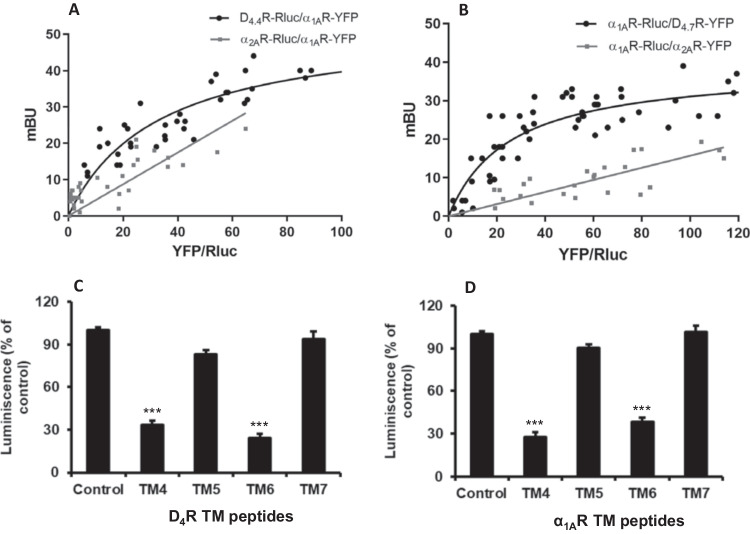
BRET experiments on heteromerization of α_1A_R with D_4.4_R and D_4.7_R. **A** HEK-293 T cells were transfected with a constant amount of D_4.4_ R-Rluc cDNA (0.11 μg) or α_2A_R-Rluc cDNA (0.008 μg), and with increasing amounts of α_1A_R-YFP cDNA (0.01 to 1.8 μg). **B** HEK-293 T cells were transfected with a constant amount of α_1A_R-Rluc cDNA (0.25 μg) and with increasing amounts of D_4.7_R-YFP cDNA (0.15 to 2.5 μg) or α_2A_R-YFP cDNA (0.2–2 μg). The relative amount of BRET is given as a function of 100 the ratio between the fluorescence of the acceptor (YFP) and the luciferase activity of the donor (Rluc). BRET is expressed as mili BRET units (mBU) of 3 to 6 different experiments. **C, D** Effect of TM peptides on α_1A_R-D_4_R heteromerization by BiLC. The figure shows the quantification of luminescence due to Rluc complementation in HEK-293 cells co-expressing α_1A_R-cRluc and D_4.4_R-nRluc in the absence (control) or presence of TM4, TM5, TM6 and TM7 peptides of D_4_R (**C**) and α_1A_R (**D**). Cells were treated for 4 h with vehicle or with the corresponding TM peptide (2 μM) before performing the complementation assay. Luminescence values (in percentage of control) represent means ± S.E.M. from 8 different experiments. The luminescence control values (without interfering peptides) were always between 120.000 and 150.000 relative luminescence units. Statistical differences in luminescence values were calculated by one-way ANOVA followed by Dunnett’s post-hoc test; ***: *P* < 0.001, *versus* control

The possible heteromerization of D_4.4_R and D_4.7_R human polymorphic variants with α_1A_R was first explored using the BRET biophysical approach. In this technique the bioluminescent donor (Rluc) and acceptor (YFP) are fused to the two putatively interacting receptors and BRET occurs when they are in very close proximity. A saturation curve indicates a specific interaction while a straight line indicates a random-collision non-specific interaction. The experiments were performed in HEK-293 T cells transiently co-transfected with the cDNA of one receptor fused to Rluc and increasing amounts of cDNA encoding the other receptor fused with YFP. Saturation BRET curves were obtained with D_4.4_R-Rluc and α_1A_R-YFP, and with α_1A_R-Rluc and D_4.7_R-YFP (Figs. 1A and B). The BRET_max_ obtained for the D_4.4_R-α_1A_R pair was 53 ± 5 mBU (in mean ± S.D. of milliBRET units, n = 4), and a significantly lower signal was obtained for the D_4.7_R-α_1A_R pair (39 ± 3 mBU, n = 6; non-paired *t* test: p < 0.001), while BRET_50_ values obtained for both pairs were not significantly different: 33 ± 7 and 25 ± 5 (in mean ± S.D.) for D_4.4_R-α_1A_R and D_4.7_R-α_1A_R, respectively. In contrast, linear plots were obtained in cells transfected with both combinations of fusion proteins of the α_2A_R-α_1A_R pair (Figs. 1A and B). These results could indicate a reduced ability of D_4.7_R to form heteromers with α_1A_R, as also suggested for D_2_R-D_4.7_R and α_2A_R-D_4.7_R heteromers, as compared with D_4.4_R [4, 6], although a reduced BRET between the intracellularly localized Rluc and YFP due to a hindrance effect related to the large 3IL of D_4.7_R could also be involved.

BiLC experiments with α_1A_R-cRluc and D_4.4_R-nRluc also demonstrate a significant proximity of both receptors, compatible with α_1A_R-D_4.4_R heteromerization, which was significantly reduced with the incubation of TM4 and TM6 peptides but not with TM5 or TM7 peptides of either D_4_R and α_1A_R (Figs. 1C and D). These results indicate that TM4 and TM6 of both receptors form part of the heteromeric interface. The corresponding TM peptides can then be used as heteromer-disrupting tools to disclose the pharmacological properties of α_1A_R-D_4_R heteromers and their presence in native tissues.

### Differences in D_4.4_R and D_4.7_R-Mediated G Protein Activation upon α_1A_R Co-Expression

**Fig. 2:**
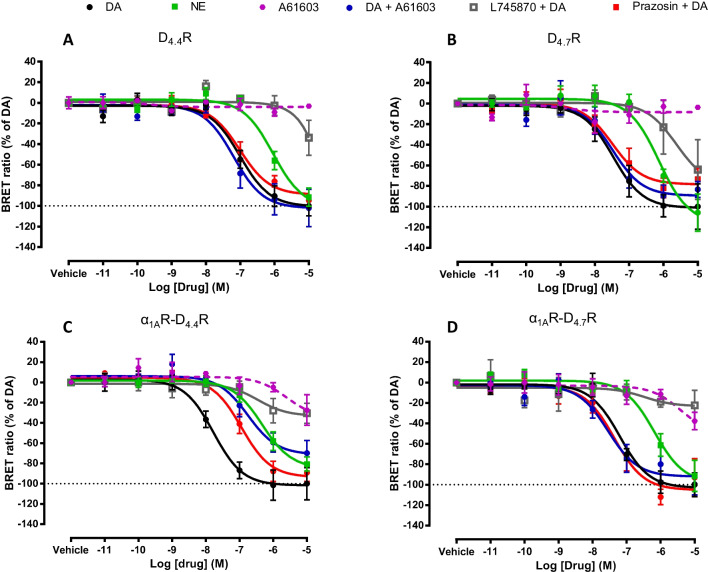
BRET experiments on ligand-induced D_4.4_R and D_4.7_R-mediated G_i_ protein activation with and without α_1A_R co-expression*.* Concentration–response experiments of dopamine (DA), norepinephrine (NE) and the α_1A_R agonist A61603 in the presence of A61603 (1 µM), the D_4_R antagonist L745870 (1 µM) or the α_1A_R antagonist prazosin (1 µM) in HEK-293 T cells transiently transfected with G_αi1_-Rluc, G_γ2_-YFP, non-fused G_β1_ and D_4.4_R (**A**), D_4.7_R (**B**), D_4.4_R plus α_1A_R (**C**) or D_4.7_R plus α_1A_R (**D**). Ligand-induced changes in BRET values were measured as described in Material and Methods. BRET values in the absence of ligands were subtracted from the BRET values for each agonist concentration. Data from all the experiments per treatment were fitted to a sigmoidal dose–response function by nonlinear regression analysis per experiment and represent means ± S.E.M. (n = 3–8, performed in triplicate) (see Table 1 for EC_50_ and E_max_ values and statistical analysis)

HEK-293 T cells were co-transfected with D_4.4_R or D_4.7_R, the G_α_ subunit of the G_i_ protein (G_αi1_) fused to Rluc, the G_γ2_ subunit fused to YFP and non-fused G_β1_ subunit, without or with co-transfection with non-fused α_1A_R. Gi protein activation was analyzed as changes in the BRET signal induced by the endogenous agonists, dopamine and norepinephrine, in the absence and presence of the D_4_R antagonist L745870, or α_1A_R ligands, the α_1A_R agonist A61603 and the α_1A_R antagonist prazosin. The effect of A61603 alone was also analyzed. The antagonists were administered 10 min before the agonists. Data were fitted to sigmoidal concentration–response curves and EC_50_ and E_max_ values were deduced. As expected, in the absence of α_1A_R, D_4.4_R and D_4.7_R showed the same pharmacological profile, with similar EC_50_ and E_max_ values for dopamine and similar values for norepinephrine, which showed about 10 times less potency and the same efficacy than dopamine (Figs. 2A and B and Table 1), as previously described [14]. Also as expected, the selective α_1A_R agonist A61603 did not produce any effect and did not modify the response to dopamine, and the selective D_4_R antagonist L745870, but not the α_1A_R antagonist prazosin, antagonized the effect of dopamine (Figs. 2A and B and Table 1). Interestingly, co-transfection with α_1A_R did not modify this pharmacological profile for D_4.7_R, but it did for the D_4.4_R. In this case, the two α_1A_R ligands, A61603 and prazosin, promoted a significant shift to the right of the dopamine concentration–response curve, with a significant decrease in the EC_50_ values (of about 8 times), strongly suggestive of selective negative allosteric modulations by α_1A_R ligands, agonists or antagonists, of the Gi protein activating effect of dopamine in the α_1A_R-D_4.4_R heteromer (Figs. 2C and D and Table 1). Apart from the *negative crosstalk* shown with agonists of both receptors, the most demonstrative results of a dependence on α_1A_R-D_4.4_R heteromerization are those showing *cross-antagonism*, the ability of a selective antagonist of a GPCR to counteract the activation of another molecularly different GPCR.

**Table 1:** Parameters of BRET experiments on ligand-induced D_4.4_R and D_4.7_R-mediated G_i_ protein activation with and without α_1A_R co-expression

G_α__i1_	DOPAMINE		NOREPINEPHRINE		A61603 + DOPAMINE		PRAZOSIN + DOPAMINE	
	EC_50_(nM)	E_max_(%)	EC_50_(nM)	E_max_(%)	EC_50_(nM)	E_max_(%)	EC_50_(nM)	E_max_(%)
D_4.4_R	137 ± 51	100 ± 10	870** ± 255	91 ± 7	99 ± 49	102 ± 18	220 ± 87	95 ± 1.2
D_4.7_R	98 ± 60	100 ± 20	870* ± 350	86 ± 17	42 ± 15	91 ± 7	89 ± 74	83 ± 10
α_1A_R-D_4.4_R	20 ± 6.7	100 ± 16	412*** ± 64	85 ± 17	152* ± 64	70 ± 12	163* ± 42	88 ± 10
α_1A_R-D_4.7_R	60 ± 10	100 ± 11	613** ± 19	91 ± 15	43 ± 12	100 ± 10	40 ± 9	108 ± 18

Potency (EC_50_ values, in nM) and relative efficacy (E_max_ values, as % of dopamine) from G_i_-protein activation BRET experiments, as shown in Fig. 2. EC_50_ and E_max_ values per experiment were obtained from a sigmoidal concentration–response function adjusted by non-linear regression analysis and are expressed as means ± S.E.M. of 3 to 8 experiments per treatment performed in triplicate. Statistical differences in EC_50_ and E_max_ values between different treatments in cells with the same transfected receptors were analyzed by one-way ANOVA, followed by Dunnett’s post hoc test; *, ** and ***: P < 0.05, P < 0.01 and P < 0.001, respectively, *versus* dopamine treatment

### Differences in α_1A_R-Mediated G Protein Activation Upon Co-Expression with D_4.4_R or D_4.7_R

Classically, α_1A_R is coupled to the G_αq/11_ protein family, but it has also been shown to couple to the G_αs_ protein-cAMP signaling pathway [23, 24]. We therefore analyzed both G protein subtypes when studying α_1A_R-mediated G protein activation in BRET experiments. HEK-293 T cells were co-transfected with α_1A_R, the G_α_ subunit of the G_q_ or G_s_ proteins (G_αq_ or G_αs_) fused to Rluc, the G_γ2_ subunit fused to YFP and non-fused G_β1_ subunit, without or with co-transfection with D_4.4_R or D_4.7_R. G_q_ or G_s_ protein activation was analyzed as changes in the BRET signal induced by endogenous agonists norepinephrine and dopamine, the selective α_1A_R agonist A61603 and the selective D_4_R agonist A412997. A61603 was then used to analyze its possible interactions with D_4_R ligands, since, differently from norepinephrine, it did not promote significant D_4_R activation (see above). The effect of A61603 was then also analyzed in the presence of A412997, L745870 and prazosin. The antagonists were administered 10 min before the agonists.

**Fig. 3:**
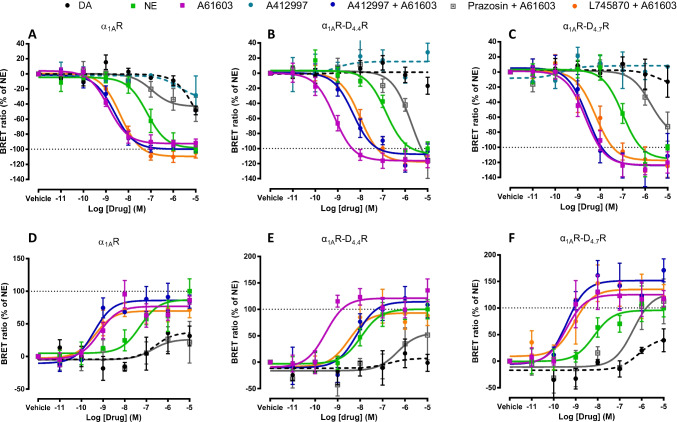
BRET experiments on ligand-induced α_1A_R-mediated G_q_ and G_s_ protein activation with and without D_4.4_R or D_4.7_R co-expression*.* Concentration–response experiments of dopamine (DA), norepinephrine (NE), the D_4_R agonist A412997 or the α_1A_R agonist A61603 alone or in the presence of A412997 (1 µM), the α_1A_R antagonist prazosin (1 µM) or the D_4_R antagonist L745870 (1 µM) in HEK-293 T cells transiently transfected with G_αq_-Rluc (**A-C**) G_αs_-Rluc (**D-F**), G_γ2_-YFP, non-fused G_β1_ and α_1A_R (**A, D**), α_1A_R plus D_4.4_R (**B, E**) or α_1A_R plus D_4.7_R (**C, F**). Ligand-induced changes in BRET values were measured as described in Material and Methods. BRET values in the absence of ligands were subtracted from the BRET values for each agonist concentration. Data from all the experiments per treatment were fitted to a sigmoidal dose–response function by nonlinear regression analysis per experiment and represent means ± S.E.M. (n = 3–8, performed in triplicate) (see Tables 2 and 3 for EC_50_ and E_max_ values and statistical analysis)

The changes in BRET values can be positive or negative depending on the G_α_ subtype as well as on the position and orientation of the inserted Rluc [25]. In the present experiment, G_i1_ and G_q_ activation produced a decrease in BRET values whereas G_s_ activation produced an increase in the BRET signal (Fig. 3). In the absence of D_4_Rs, norepinephrine promoted a significant G_q_ and G_s_ activation, while dopamine was mostly inefficient (less than 50% as compared to norepinephrine, at the highest 10 μM concentration), which did not allow reliable EC_50_ calculations, indicating that it should be at least two orders of magnitude higher than the EC_50_ values for norepinephrine (Figs. 3A and D). In both cases, A61603 was more potent and as effective as norepinephrine, and the effect of A61603 was counteracted by prazosin and not modified by A412997 or L745870 (Figs. 3A and D and Tables 2 and 3). The same pharmacological profile was observed with co-transfection with D_4.7_R (Figs. 3C and F), while upon co-transfection with D_4.4_R, both D_4_R ligands promoted a significant shift to the right of the A61603 concentration–response curves, with an increase of more than ten times in the EC_50_ values (Figs. 3B and E and Tables 2 and 3). This is also strongly suggestive of reciprocal selective negative allosteric modulations, by which D_4_R agonists or antagonists counteract the Gq and Gs protein activating effect of the α_1A_R agonist A61603 in the α_1A_R-D_4.4_R heteromer.

**Table 2:** Parameters of BRET experiments on ligand-induced α_1A_R-mediated G_q_ protein activation with and without D_4.4_R or D_4.7_R co-expression

G_αq_	NOREPINEPHRINE		A61603		A61603 + A412997		L745870 + A61603	
	EC_50_(nM)	E_max_(%)	EC_50_(nM)	E_max_(%)	EC_50_(nM)	E_max_(%)	EC_50_(nM)	E_max_(%)
α_1A_R	200* ± 120	100 ± 5	2.8 ± 1.6	92 ± 5	2.7 ± 1.1	99 ± 5	4.8 ± 1.1	105 ± 7
α_1A_R-D_4.4_R	140** ± 49	100 ± 9	0.88 ± 0.25	117 ± 8	10* ± 3	103 ± 10	16* ± 6	118 ± 7
α_1A_R-D_4.7_R	214* ± 69	100 ± 3	3 ± 1.5	120 ± 14	2.7 ± 0.4	111 ± 30	7.4 ± 4	125 ± 15

Potency (EC_50_ values, in nM) and relative efficacy (E_max_ values, as % of norepinephrine) from G_q_-protein activation BRET experiments, as shown in Fig. 3. EC_50_ and E_max_ values per experiment were obtained from a sigmoidal concentration–response function adjusted by non-linear regression analysis and are expressed as means ± S.E.M. of 3 to 8 experiments per treatment performed in triplicate. Statistical differences in EC_50_ and E_max_ values between different treatments in cells with the same transfected receptors were analyzed by one-way ANOVA, followed by Dunnett’s post hoc test; * and **: P < 0.05, P < 0.01, respectively, *versus* A61603 treatment

**Table 3:** Parameters of BRET experiments on ligand-induced α_1A_R-mediated G_s_ protein activation with and without D_4.4_R or D_4.7_R co-expression

G_α__s_	NOREPINEPHRINE		A61603		A61603 + A412997		L745870 + A61603	
	EC_50_(nM)	E_max_(%)	EC_50_(nM)	E_max_(%)	EC_50_(nM)	E_max_(%)	EC_50_(nM)	E_max_(%)
α_1A_R	58* ± 10	100 ± 19	1.8 ± 0.6	86 ± 6	3.8 ± 0.7	84 ± 10	1.5 ± 1.2	72 ± 12
α_1A_R-D_4.4_R	19** ± 11	100 ± 18	0.3 ± 0.08	132 ± 21	11* ± 6	106 ± 24	2.7* ± 0.28	50 ± 45
α_1A_R-D_4.7_R	80** ± 57	100 ± 17	1.1 ± 0.5	117 ± 14	0.63 ± 0.15	140 ± 30	1.3 ± 0.6	120 ± 23

Potency (EC_50_ values, in nM) and relative efficacy (E_max_ values, as % of norepinephrine) from G_s_-protein activation BRET experiments, as shown in Fig. 3. EC_50_ and E_max_ values per experiment were obtained from a sigmoidal concentration–response function adjusted by non-linear regression analysis and are expressed as means ± S.E.M. of 3 to 7 experiments per treatment performed in triplicate. Statistical differences in EC_50_ and E_max_ values between different treatments in cells with the same transfected receptors were analyzed by one-way ANOVA, followed by Dunnett’s post hoc test; *, ** and ***: P < 0.05, P < 0.01 and P < 0.001, respectively, *versus* A61603 treatment

### Different Modulation of Adenylyl Cyclase and Calcium Signaling in α_1A_R-D_4.4_R and α_1A_R-D_4.7_R Cells

**Fig. 4:**
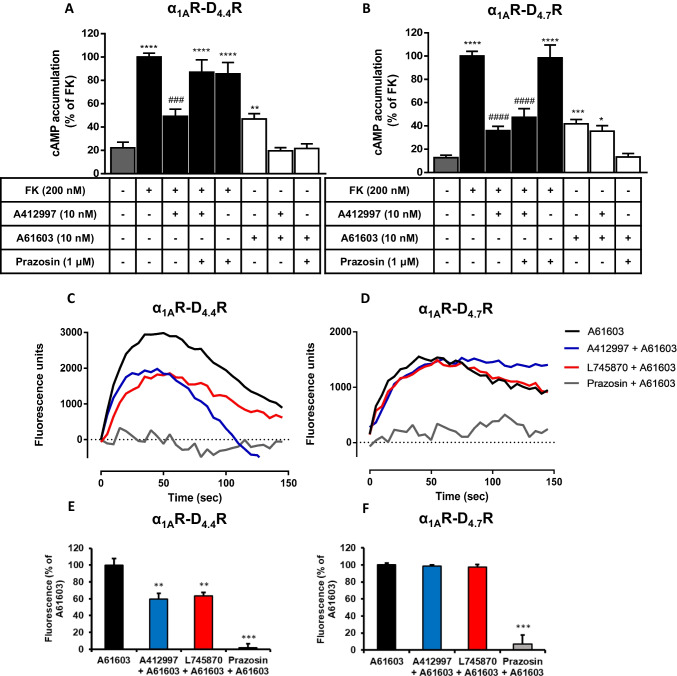
Adenylyl cyclase and calcium signaling in α_1A_R-D_4.4_R and α_1A_R-D_4.7_R cells. **A, B** cAMP formation in tetracycline-inducible HEK-293 T cells expressing D_4.4_R (**A**) or D_4.7_R (**B**) and transiently co-transfected with α_1A_R. cAMP formation was induced by forskolin (FK, 200 nM; black bars) or with the α_1_R agonist A61603 (10 nM; white bars) in the presence or absence of the D_4_R agonist A412997 (10 nM) or the α_1_R antagonist prazosin (1 μM). Values are means ± S.E.M. of 3 to 5 experiments, expressed as % of cAMP induced by forskolin. Statistical differences between different treatments were analyzed by one-way ANOVA, followed by Dunnett’s post hoc test; *, **, *** and ****: P < 0.05, P < 0.01, P < 0.001 and < 0.0001, respectively, *versus* basal; ^###^ and ^####^: P < 0.001 and P < 0.0001, respectively, *versus* forskolin. **C-E** Intracellular calcium mobilization in HEK-293 T cells transiently co-transfected with GCaMP6, D_4.4_R and α_1A_R (**C, E**) or D_4.7_R and α_1A_R (**D, F**). Cells were activated with 20 nM of A61603 (black), co-activated with A61603 and A412997 (blue) or pre-treated with 60 nM of prazosin (grey) or L745870 (red) before A61603 activation. **C, D** Representative experiments showing the intracellular calcium release curves over time. **E, F** Values are means ± S.E.M. of 4 experiments, expressed as % of maximal effect of A61603. Statistical differences between different treatments were analyzed by one-way ANOVA, followed by Dunnett’s post hoc test; ** and ***: P < 0.01 and P < 0.001, respectively

The consequence of the specific allosteric modulations between α_1A_R and D_4_R ligands on G protein activation demonstrated in cells co-expressing α_1A_R and D_4.4_R were analyzed at the level of G protein-dependent signaling. First, on adenylyl cyclase signaling, with cAMP accumulation experiments, where activation of G_s_ proteins increases cAMP formation, while activation of G_i_ proteins inhibits forskolin-induced cAMP accumulation. These experiments were performed in previously characterized inducible D_4.4_R and D_4.7_R cell lines [4] co-transfected with α_1A_R. The D_4_R agonist A412997 (10 nM) significantly inhibited forskolin-induced cAMP accumulation, and the α_1A_R agonist A61603 (10 nM) promoted a discrete but significant cAMP accumulation (Figs. 4A and B). In both α_1A_R-D_4.4_R and α_1A_R-D_4.7_R cells, prazosin (1 μM) did not modify the effect of forskolin and counteracted A61603-induced cAMP accumulation. In α_1A_R-D_4.4_R cells, but not in α_1A_R-D_4.7_R cells, prazosin also counteracted the effect of A412997, and A412997 counteracted A61603-induced cAMP accumulation (Figs. 4A and B).

Intracellular calcium mobilization was then used as a correlative measure of Gq activation. Both in α_1A_R-D_4.4_R cells and α_1A_R-D_4.7_R cells, A61603 promoted a clear increase in the intracellular calcium signal, which was counteracted by prazosin. Only in α_1A_R-D_4.4_R cells, the effect of A61603 was significantly decreased by the D_4_R ligands, A412997 and L745870 (Figs. 4C-E). In summary, the G protein activation and the G protein-dependent signaling experiments demonstrate functional differences between D_4.4_R and D_4.7_R that depend on the co-expression with the α_1A_R. Specifically the D_4.4_R variant determines the appearance of reciprocal negative crosstalk and cross-antagonism between α_1A_R and D_4.4_R cells, which are pharmacological properties that are often simultaneously disclosed by GPCR heteromers [19, 22]. The negative results obtained in α_1A_R-D_4.7_R cells, so far would indicate the lack of allosteric interactions in the α_1A_R-D_4.7_R heteromer.

### Similar Modulation of β-arrestin Recruitment and MAPK Signaling in α_1A_R-D_4.4_R and α_1A_R-D_4.7_R Cells

Before excluding the existence of allosteric interactions in the α_1A_R-D_4.7_R heteromer, we also studied G protein-independent signaling. Thus, in previous studies we found that allosteric modulations in GPCR heteromers can have functional selectivity, i.e., selectivity for a signaling pathway, such as for a G protein-dependent or G protein-independent pathway. For instance, in the dopamine D_1_ receptor (D_1_R)-D_3_R heteromer, there is a specific G protein-independent, β-arrestin-mediated synergistic interaction between D_1_R and D_3_R agonists [20]. The presence of these potentially independent allosteric interactions can also be modulated by different cellular mechanisms, such as intracellular calcium levels, as reported for the A_2A_R-D_2_R heteromer [26]. HEK-293 T cells were co-transfected with non-fused D_4.4_R or D_4.7_R, α_1A_R fused to YFP and β-arrestin-2 fused to Rluc. The β-arrestin recruitment is then quantified as changes in BRET signal induced by increasing concentrations of ligands. Importantly, this assay should constitute an additional method to reveal α_1A_R-D_4.7_R heteromers. Thus, the BRET detection of β-arrestin-2-Rluc recruitment by D_4_R agonists with α_1A_R fused to YFP implies a very significant proximity between both receptors, a β-arrestin recruitment by the GPCR heteromer. Similarly, interactions between D_4_R and α_1A_R ligands should imply allosteric interactions within the α_1A_R-D_4.7_R heteromer.

**Fig. 5:**
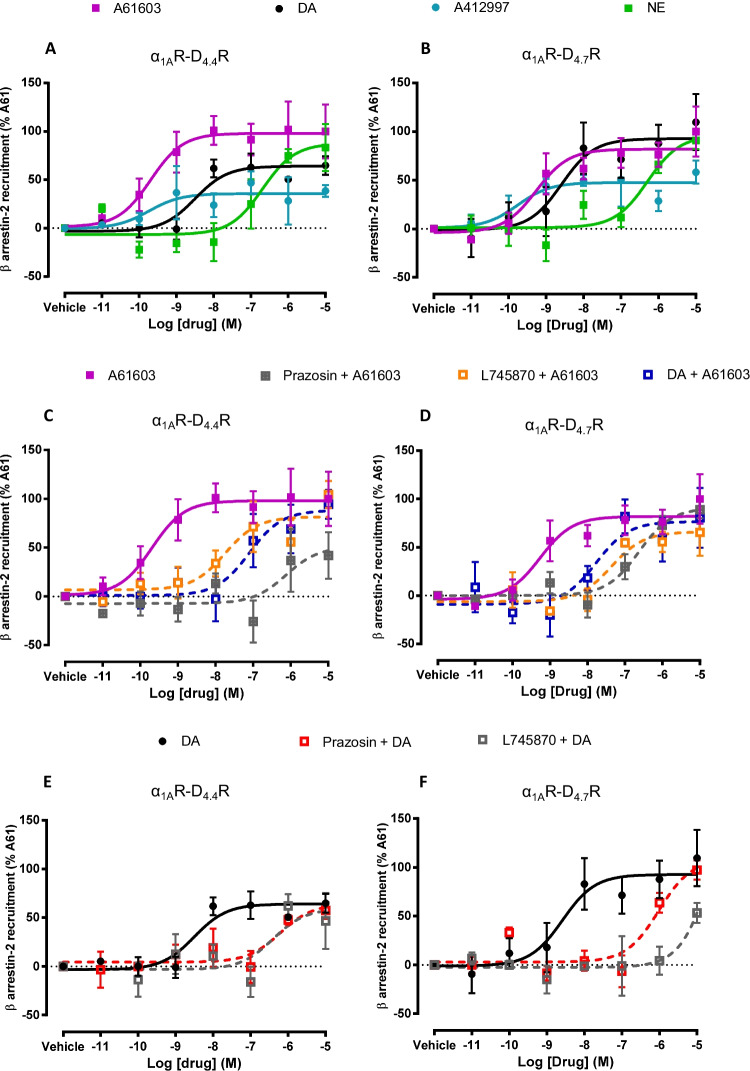
β-arrestin-2 recruitment-BRET experiments in HEK-293 T cells transiently transfected with D_4.4_R or D_4.7_R, α_1A_R-YFP and β-arrestin-2-Rluc. **A, B **Concentration–response curves induced by endogenous ligands dopamine (DA) or norepinephrine (NE), the α_1A_R agonist A61603 or the D_4_R agonist A412997 alone. **C, D **Concentration response-curve of A61603 in the presence of the α_1A_R antagonist prazosin (1 μM), the D_4_R antagonist L745870 (1 μM) or dopamine (10 nM). **E, F **Concentration response-curve of dopamine in the presence of the α_1A_R antagonist prazosin (1 μM) or the D_4_R antagonist L745870 (1 μM). After 7 min of drug exposure, BRET was measured as described in Materials and Methods. BRET values in the absence of ligands were subtracted from the BRET values for each condition. Data from all the experiments per treatment were fitted to a sigmoidal dose–response function by nonlinear regression analysis per experiment and represent means ± S.E.M. (n = 3–8, performed in triplicate) and are shown as a percentage of A61603 3 activation (see Table 4 for EC_50_ and E_max_ values and statistical analysis)

In fact, both in α_1A_R-D_4.4_R and α_1A_R-D_4.7_R cells, not only norepinephrine and A61603 promoted β-arrestin-2 recruitment, but also dopamine and the D_4_R agonist A412997 (Figs. 5A and B and Table 4), indicating the presence of functional α_1A_R-D_4.4_R and α_1A_R-D_4.7_R heteromers. Importantly, a qualitative different profile could be observed between both heteromers, with a lower relative efficacy of dopamine *versus* norepinephrine in the α_1A_R-D_4.4_R heteromer. In both heteromers, the potency of dopamine was higher than the one obtained in the G protein activation experiments and its relative potency *versus* norepinephrine was about two orders of magnitude. Importantly, negative crosstalk and cross-antagonism between α_1A_R and D_4._R ligands could be observed in both α_1A_R-D_4.4_R and α_1A_R-D_4.7_R heteromers. In both cases, the concentration response-curve of A61603 was substantially shifted to the right, not only with prazosin, but also with dopamine or L745870 (Figs. 5C and D and Table 4). Also, for both α_1A_R-D_4.4_R and α_1A_R-D_4.7_R heteromers, the concentration response-curve of dopamine was substantially shifted to the right, not only with L745870, but also with prazosin (Figs. 5E and F and Table 4).

**Table 4:** Parameters of BRET experiments on ligand-induced β-arrestin-2 recruitment in cells expressing α_1A_R and D_4.4_R or D_4.7_R

	α_1A_R-D_4.4_R		α_1A_R-D_4.7_R	
	EC_50_(nM)	E_max_(%)	EC_50_(nM)	E_max_(%)
DOPAMINE	3 ± 0.9	64 ± 9.6	3.8 ± 1.5	110 ± 29
NOREPINEPRHINE	215^###^ + 55	83 ± 24	600^###^ + 120	91 ± 9
A61603	1.1 ± 0.9	100 ± 28	1.1 ± 0.8	100 ± 26
A412997	30 ± 19	39 ± 6	11 ± 10	58 ± 11
A61603 + DOPAMINE	56^###^ ± 14	94 ± 15	30^###^ ± 11	80 ± 30
L745870 + DOPAMINE	315* ± 60	40 ± 21	1600* ± 770	53 ± 10
PRAZOSIN + DOPAMINE	711* ± 196	58 ± 8.7	1100* ± 250	97 ± 9
L745870 + A61603	14^#^ ± 4.5	104 ± 14	44^#^ ± 13	65 ± 24
PRAZOSIN + A61603	130^###^ ± 4	42 ± 23	300^###^ ± 24	89 ± 9

Potency (EC_50_ values, in nM) and relative efficacy (E_max_ values, as % of A61603) from β-arrestin-2 recruitment BRET experiments, as shown in Fig. 5. EC_50_ and E_max_ values per experiment were obtained from a sigmoidal concentration–response function adjusted by non-linear regression analysis and are expressed as means ± S.E.M. of 3 to 7 experiments per treatment performed in triplicate. Statistical differences in EC_50_ and E_max_ values between different treatments in cells with the same transfected receptors were analyzed by one-way ANOVA, followed by Dunnett’s post hoc test; *: P < 0.05, *versus* dopamine treatment; ^#^, and ^###^: P < 0.05 and P < 0.001, respectively *versus* A61603 treatment

**Fig. 6:**
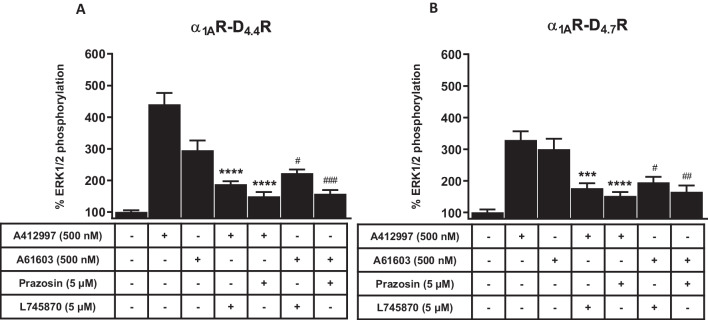
MAPK signaling in α_1A_R-D_4.4_R and α_1A_R-D_4.7_R cells. **A, B **ERK1/2 phosphorylation in HEK-293 T cells transiently transfected with α_1_R and D_4.4_R (**A**) or D_4.7_R (**B**). ERK1/2 phosphorylation was induced by the α_1_R agonist A61603 (500 nM) or the D_4_R agonist A412997 (500 nM) in the presence or absence of the α_1_R antagonist prazosin (5 μM) or the D_4_R antagonist L745870 (5 μM). The values represent the mean ± S.E.M. of quantified immunoreactive bands corresponding to ERK1 and ERK2 of 4 to 8 experiments and expressed as percentage of values from non-treated cells. Statistical differences between different treatments were analyzed by one-way ANOVA, followed by Dunnett’s post hoc test; *** and ****: P < 0.001 and P < 0.0001, respectively, *versus* A412997; ^#, ##^ and ^###^: P < 0.05, P < 0.01 and P < 0.001, respectively, *versus* A61603

We also analyzed MAPK signaling (ERK1/2 phosphorylation), which is often a G protein-independent and β-arrestin-mediated signaling, and the results paralleled those of the β-arrestin-2 recruitment. In α_1A_R-D_4.4_R and α_1A_R-D_4.7_R cells, A412997 and A61603 promoted ERK1/2 phosphorylation which, in both cases, were counteracted by both prazosin and L745870 (Figs. 6A and B). Altogether, these experiments strongly support that α_1A_Rs form functional heteromers with both D_4.4_R and D_4.7_R and that they show a differential profile in their allosteric interactions, with negative crosstalk and cross-antagonism that occur at the level of G protein-dependent and independent signaling for the α_1A_R-D_4.4_R heteromer and which are functionally selective, β-arrestin-dependent, for the α_1A_R-D_4.7_R heteromers.

### Identification of Functional α_1A_R-D_4_R Heteromers in the Rat Frontal Cortex and Striatum

**Fig. 7:**
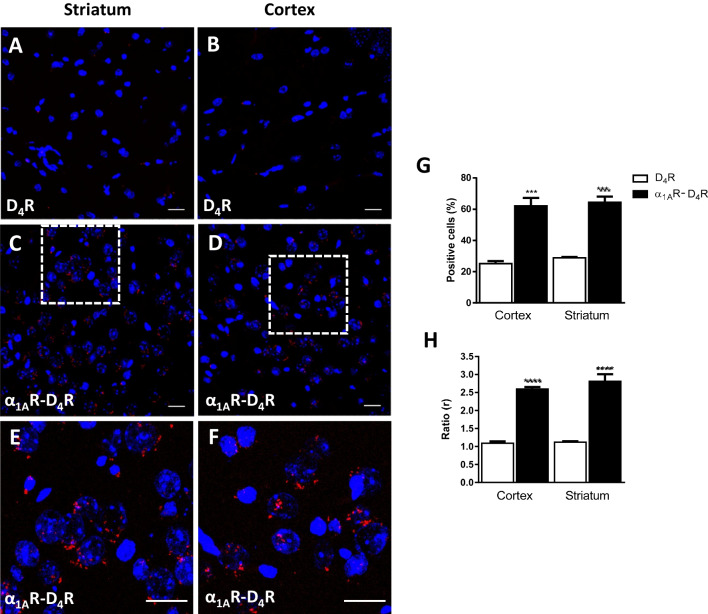
Detection of α_1a_R-D_4_R complexes in rat brain by PLA. **A, B **Absence of complexes in the absence of α_1a_R antibody (negative controls) in striatal (**A**) and cortical slices (**B**). **C, D** α_1A_R-D4R complexes observed as red around blue-coloured DAPI-stained cell nuclei in striatal (**C**) and cortical slices (**D**) and (**E, F)** cropped figures of the main C and D images.** G** Number of cells containing one or more red spots, expressed as a percentage of the total number of cells. **H** r values (number of red spots/cells containing spots). Data are the mean ± S.E.M. of counts of 3 different experiments. Statistical differences versus negative control were analyzed by one‐way ANOVA followed by Dunnett’s post hoc test (***P < 0.001; ****P < 0.0001). Scale bar: 20 μm

The possible existence of α_1A_R-D_4_R heteromers in the rat brain, in the frontal cortex and striatum, was first analyzed by using the proximity ligation assay (PLA). PLA requires that both receptors be close enough (< 40 nm) to allow the two different antibody-based probes to ligate (see Materials and Methods). If the receptors are forming complexes, a punctate fluorescent signal can be detected by confocal microscopy. Red dots were detected surrounding DAPI-positive nuclei in striatal and cortical slices (Figs. 7C, D, E and F). The number of apparent cells with red dots was significantly higher than the number of dots from slices treated only with one primary antibody and both secondary antibodies (negative controls) (Figs. 7A, B, G and H). These results indicate the existence of complexes of α_1A_R and D_4_R in the rat brain, compatible with α_1A_R-D_4_R heteromers. Although the experiments did not allow to identify if the dots are preferentially expressed presynaptically in nerve terminals establishing contact with cell bodies, or postsynaptically in the somatodendritic area, they should be expected to mainly label cortico-striatal glutamatergic terminals, where both receptors can mostly be co-localized (see Introduction and Discussion).

**Fig. 8:**
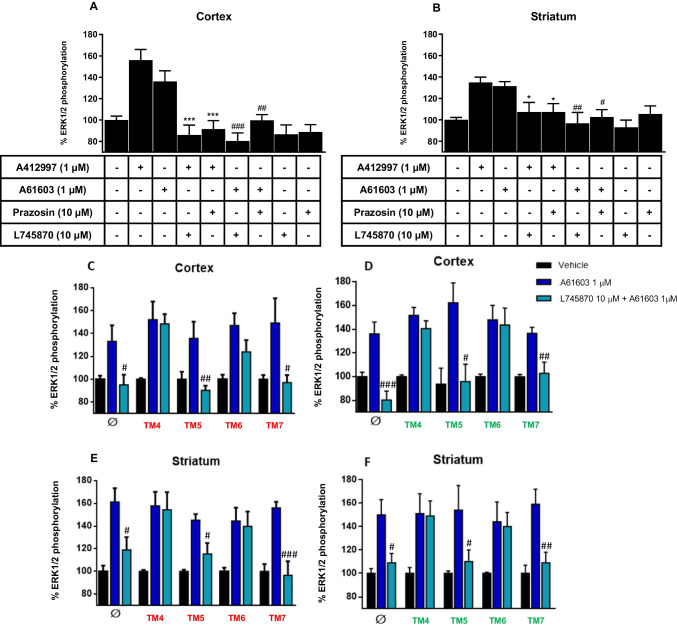
MAPK signaling in rat brain. **A, B** ERK1/2 phosphorylation in rat cortical (**A**) or striatal slices (**B**). ERK1/2 phosphorylation was induced by the α_1_R agonist A61603 (1 μM) or the D_4_R agonist A412997 (1 μM) in the presence or absence of the α_1_R antagonist prazosin (10 μM) or the D_4_R antagonist L745870 (10 μM). **C-F** Effect of TM peptides of α_1A_R and D_4_R. Slices were not pretreated (Ø) or were pre-treated for 4 h with 4 μM of TM4-TM7 peptides of α_1A_R (labelled in red) or D_4_R (labelled in green). Slices were not stimulated (vehicle), stimulated for 10 min with A61603 or pre-treated for 20 min with 10 µM of L745870 before A61603 treatment. The values represent the mean ± S.E.M. of quantified immunoreactive bands corresponding to ERK1 and ERK2 of 4 experiments and expressed as percentage of values from non-treated slices. Statistical differences between different treatments were analyzed by one-way ANOVA, followed by Dunnett’s post hoc test; * and ***: P < 0.05 and P < 0.001, respectively, *versus* A412997; ^#, ##^ and ^###^: P < 0.05, P < 0.01 and P < 0.001, respectively, *versus* A61603

We then used a more functional but also more demonstrative method to identify α_1A_R-D_4_R heteromers in rat cortical and striatal slices, based on the identification of a pharmacological property of the heteromer (biochemical fingerprint) and on its specific disruption by synthetic peptides that specifically disrupt α_1A_R-D_4_R heteromers (demonstrated by BiLC experiments, Figs. 1C and D). ERK1/2 phosphorylation induced by the D_4_R agonist A412997 and the α_1A_R agonist A61603 was analyzed in slices from rat frontal cortex or striatum, incubated in the absence or presence of the D_4_R antagonist L745870 or the α_1A_R antagonist prazosin, and incubated in the absence or presence of α_1A_R-D_4_R heteromer-disrupting peptides. In the absence of peptides, both in cortical and striatal slices, A412997 (1 μM) and A61603 (1 μM) promoted ERK1/2 phosphorylation, which, for both agonists, was significantly counteracted by L745870 and prazosin at a concentration (10 μM) that did not produce a significant effect versus basal values (Figs. 8A and B). Importantly, both in cortical and striatal slices, the L745870-mediated cross-antagonism of A61603 was significantly and selectively counteracted by the TM peptides of both α_1A_R and D_4_R that disrupted α_1A_R-D_4_R heteromerization (TM4 and TM6, but not TM5 and TM7) (Figs. 8C-F). Altogether, these experiments demonstrate that a significant proportion of frontal cortical and striatal α_1A_R and D_4_R form functional α_1A_R-D_4_R heteromers.

## Discussion

The present study demonstrates the ability of the D_4_R to form functional heteromers with α_1A_R in the brain, adding to the list of heteromers of D_4_R with other adrenoceptors, which includes α_1B_R and β_1_R in the pineal gland and α_2A_R in the frontal cortex [1]. In addition, it also adds to the studies that showed that the functional and pharmacological differences between the D_4_R polymorphic variants, more specifically between D_4.4_R and D_4.7_R, can be disclosed upon heteromerization with other GPCRs, such as α_2A_R and D_2_R [4–6, 14]. It has been suggested that α_2A_R-D_4_R and D_2_R-D_4_R, respectively localized in the cortical perisomatic region and in the striatal nerve terminals of pyramidal neurons, exert a key modulatory role of cortico-striatal glutamatergic neurotransmission [1]. This modulatory role is different depending on the D_4_R variant, which depends on the existence of different allosteric modulations in the respective heteromers, with the presence of D_4.7_R promoting a gain of function of the D_2_R-mediated dopaminergic and α_2A_R-mediated noradrenergic inhibitory control of cortico-striatal glutamatergic transmission [1].

Nevertheless, α_1A_R-D_4_R heteromers represent a significant population of functional interacting α_1A_Rs and D_4_Rs localized in the rat frontal cortex and in the striatum, as demonstrated with PLA and MAPK activation experiments. In the striatum, the demonstration of α_1A_R-D_4_R heteromers implies their preferential role in the modulation of striatal glutamate release, in view of the preferential striatal localization of both receptors in striatal glutamatergic terminals [15, 16]. In this case, as discussed below, it is the presence of the D_4.4_R, and not the D_4.7_R, what might result on a gain of function of the α_1A_R-mediated noradrenergic stimulatory control of cortico-striatal glutamatergic neurotransmission.

The analysis of G protein activation and signaling in cells expressing α_1A_Rs and D_4.4_Rs or D_4.7_Rs, also demonstrated pharmacological differences between both polymorphic variants that depend on heteromerization with α_1A_Rs. Differently to the α_1A_R-D_4.4_R heteromer, the allosteric interactions in the α_1A_R-D_4.7_R heteromer were functionally selective and could only be observed in experiments of G protein-independent signaling (β-arrestin recruitment and MAPK activation). Thus, significant allosteric interactions could also be observed in the α_1A_Rs-D_4.4_R heteromer in experiments of G protein activation and G protein-dependent signaling (adenylate cyclase activity or intracellular calcium mobilization). When present, the allosteric interactions between α_1A_R and D_4.4_R ligands were reciprocal and antagonistic, and agonists or antagonists of one of the receptors negatively modulated the result of the activation of the other molecularly different receptor (negative crosstalk or cross-antagonism).

The α_1A_Rs-D_4.4_R heteromer is functionally like other GPCR heteromers constituted by two molecularly different GPCRs separately coupled to stimulatory and inhibitory G proteins, which promote neuronal activation and inhibition, respectively. For instance, the A_2A_R-D_2_R heteromer and D_1_R-D_3_R heteromers, with a tetrameric structure that allows the simultaneous coupling of G_s_ to an A_2A_R or D_1_R homodimer and G_i_ to a D_2_R or D_3_R homodimer [20–22, 26]. In these GPCR heteromers multiple and reciprocal G protein-dependent and independent allosteric interactions can be identified which can be subjected to differential control by different exogenous ligands or intracellular messengers. The output of these integrative devices will therefore depend on the final integrated signaling of the respective G_s_ and G_i_ targeted plasma membrane and intracellular effectors. In fact, some plasma membrane effectors, such as adenylyl cyclase and GIRKs oligomerize with GPCR heteromers, forming part of G protein-coupled-effector macromolecular membrane assemblies (GEMMA; [27]).

Irrespective of their coupling to stimulatory or inhibitory G proteins, activation of most GPCRs leads to MAPK signaling, which is often dependent on β-arrestin recruitment. There is still a significant lack of understanding of the functional neuronal and behavioral correlates of the isolated or combined activation or inhibition of the different GPCR-targeted cellular effectors. Nevertheless, it is generally accepted that the most immediate responses of plasma membrane effectors, which mediates early changes in neuronal excitability and neurotransmitter release, are mediated by their direct interaction with G protein subunits [27]. On the other hand, MAPK activation mediates more protracted gene-expression-mediated effects. It should therefore be expected that the lack of G protein-dependent allosteric interactions in the α_1A_R-D_4.7_R heteromer would determine significant functional neuronal and behavioural differences, as compared with the α_1A_R-D_4.4_R heteromer.

According to the results from G protein activation, norepinephrine can potentially bind to α_1A_R and, with higher concentrations, to the D_4_R, while dopamine seems to need exceedingly large concentrations to bind to α_1A_R. This would not support previous suggestions about α_1A_R being a target for endogenous dopamine [28, 29]. Although the striatum is classically a main target of the dopaminergic system, its more ventral component, the shell of the nucleus accumbens (NAc), also receives a substantial noradrenergic innervation, both in rodents and humans, and significant basal concentrations of noradrenaline can be detected in this striatal compartment by microdialysis and shown to significantly increase with amphetamine administration [30–32]. Also, a seminal study by Weinshenker and colleagues showed a significant role of α_1A_R in the modulation of glutamate and secondarily dopamine release and in the locomotor activating effects of cocaine and morphine [15]. So, the present study indicates that a significant population of these striatal α_1A_Rs and D_4_Rs form functional heteromers.

The D_4.4_R and D_4.7_R polymorphic variants should determine significant differences in the integration of norepinephrine and dopamine in the ventral striatum operated by α_1A_R-D_4.4_R or α_1A_R-D_4.7_R heteromers. The separate activation of α_1A_R or D_4_R should lead to facilitation and inhibition of glutamate release, respectively, but upon norepinephrine release, the activation of α_1A_R should allosterically counteract D_4.4_R, but not D_4.7_R-mediated inhibition. We should then expect the D_4.4_R variant to provide a gain of function of the α_1A_R-mediated noradrenergic stimulatory control of frontal cortico-striatal glutamatergic neurotransmission. This could therefore imply a lower degree of cortico-striatal transmission during conditions of stress in the presence of α_1A_R-D_4.7_R as compared to α_1A_R-D_4.4_R heteromers, which would add to the lower degree of cortico-striatal transmission determined by the striatal D_2_R-D_4.7_R heteromers (maybe more prevalent in the dorsal striatum) and the cortical α_2A_R-D_4.7_R heteromers [1]. Therefore, as suggested for D_2_R-D_4.7_R and α_2A_R-D_4.7_R, the α_1A_R-D_4.7_R heteromers could also increase the vulnerability of impulse control-related neuropsychiatric disorders while it could also decrease the vulnerability of PTSD (see Introduction).

D_4.4_R and D_4.7_R confer significantly different functional and pharmacological properties to α_1A_R-D_4_R heteromers, which mediate a dopamine- and norepinephrine-dependent fine-tune modulation of the frontal cortico-striatal glutamatergic neuronal function. α_1A_R-D_4_R heteromers may explain a differential vulnerability for PTSD and the differential effect of D_4_R polymorphisms in the moderation of the impulsivity traits and their role in impulse control-related neuropsychiatric disorders, including ADHD, and more specifically, the association of D_4.7_R with impulse-control disorders.

## Data Availability

All data needed to evaluate the conclusions in this study are present in the paper.
